# Synchronous sigmoid and transverse volvulus: A case report and qualitative systematic review

**DOI:** 10.1016/j.ijscr.2020.09.027

**Published:** 2020-09-10

**Authors:** Abdourahmane Ndong, Mohamed Lamine Diao, Jacques Noel Tendeng, Adja Coumba Diallo, Philippe Manyacka Ma Nyemb, Ibrahima Konaté

**Affiliations:** Department of Surgery, Gaston Berger University, Saint-Louis, Senegal

**Keywords:** Volvulus, Sigmoid, Transverse, Colon, Simultaneous

## Abstract

**Introduction:**

Colonic volvulus is defined as a torsion of a part of the colon causing large bowel obstruction by strangulation which may lead to ischemia and then necrosis. The synchronous occurrence of a sigmoid colon and transverse colon volvulus is exceptional. We describe a case of synchronous sigmoid and transverse volvulus in a patient with a qualitative systematic review of this condition.

**Presentation of the case:**

This is a 74-year-old patient with a history of chronic constipation, who consulted for bowel obstruction. Plain abdominal radiography showed diffuse gas distension of the colon with the absence of rectal gas. An exploratory laparotomy was performed and showed sigmoid colon volvulus associated with synchronous transverse colon volvulus without bowel necrosis. A left hemicolectomy with loop colostomy was performed. The restoration of bowel continuity was done 3 weeks. The post-operative course was uneventful.

**Discussion:**

The occurrence of a simultaneous sigmoid and transverse colonic volvulus is an exceptional situation. Due to the rarity of this clinical entity, the literature concerning its description is sparse and the treatment options are poorly codified. There are no guidelines in the treatment and a tailored approach should be used for each patient.

**Conclusion:**

The dual location of strangulation makes this situation a major surgical emergency with a high risk of gangrene and septic shock. Colectomy with delayed anastomosis should be preferred in the treatment.

## Highlights

• Colonic volvulus represents the leading cause of colonic obstruction in sub-Saharan Africa.
• The sigmoid colon is the primary site of volvulus while the transverse colon is a rare condition.
• The synchronous occurrence of a sigmoid colon and transverse colon volvulus is exceptional.
• The dual location of strangulation makes it a major surgical emergency with a high risk of gangrene and septic shock.
• The literature concerning its description is sparse and the treatment options are poorly codified.

## 1 Introduction

The work has been reported in line with the SCARE criteria [1].

Colonic volvulus is defined as a torsion of a part of the colon causing large bowel obstruction by strangulation which may lead to ischemia and then necrosis [2]. It accounts for 3–5% of acute bowel obstructions and is the third leading cause of colonic obstruction worldwide after tumor obstruction and complicated sigmoid diverticulitis [3]. Besides, it represents the leading cause of colonic obstruction in sub-Saharan Africa with a prevalence of up to 69% [4].

It remains particularly common in Africa, South America, Russia, the Middle East, and Eastern Europe determining the geographic region known as the “Volvulus Belt” [2,3]. It constitutes a life-threatening emergency when diagnosis and treatment are not made early.

The sigmoid colon is the primary site of volvulus in 60–75% followed by the cecum in 25–40% of cases [3]. While the transverse colon only represents 4%, making it a rare condition [5,6]. Simultaneous volvuli represent uncommon situations. Also, the clinicals presentations more frequently described are ileo-sigmoid knot in one hand, and synchronous sigmoid with cecal volvulus [7].

The synchronous occurrence of a sigmoid colon and transverse colon volvulus is exceptional. The clinical signs are those of large bowel obstruction. Therefore, imaging does not always suggest this unusual diagnosis causing generally a per-operative diagnosis.

The dual location of strangulation makes it a major surgical emergency with a high risk of gangrene and septic shock. Due to the rarity of this clinical entity, the literature concerning its description is sparse and the treatment options are poorly codified.

Our objective is to describe a case of synchronous sigmoid and transverse volvulus in a 74-year-old patient in order to perform a qualitative systematic review of this rare condition.

## 2 Case report

**Fig. 1:**
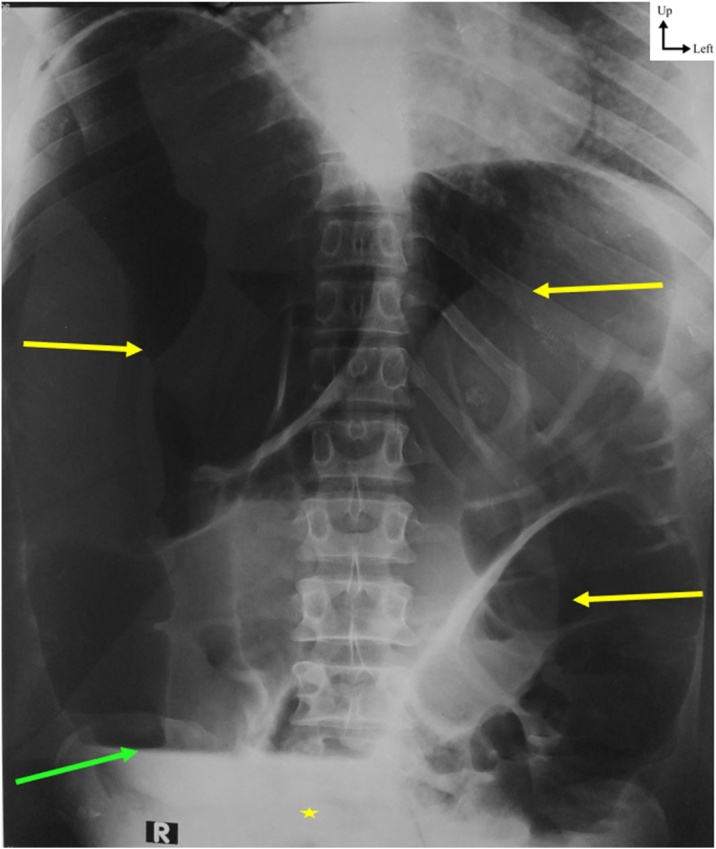
Plain abdominal radiography showing diffuse gas distension of the colon (yellow arrows) with hydro-aeric levels (green arrow) and without gas in the pelvis (yellow star).

This is a 74-year-old patient with a history of chronic constipation, who consulted for abdominal pain and vomiting evolving for 48 h. The patient had no particular personal or familial medical history. The physical examination found a good general status with physiological constants were normal. Palpation found a diffuse meteorism without tenderness. The rectal examination was unremarkable. The biology was normal. Plain abdominal radiography showed diffuse gas distension of the colon with the absence of rectal gas (Fig. 1).

**Fig. 2:**
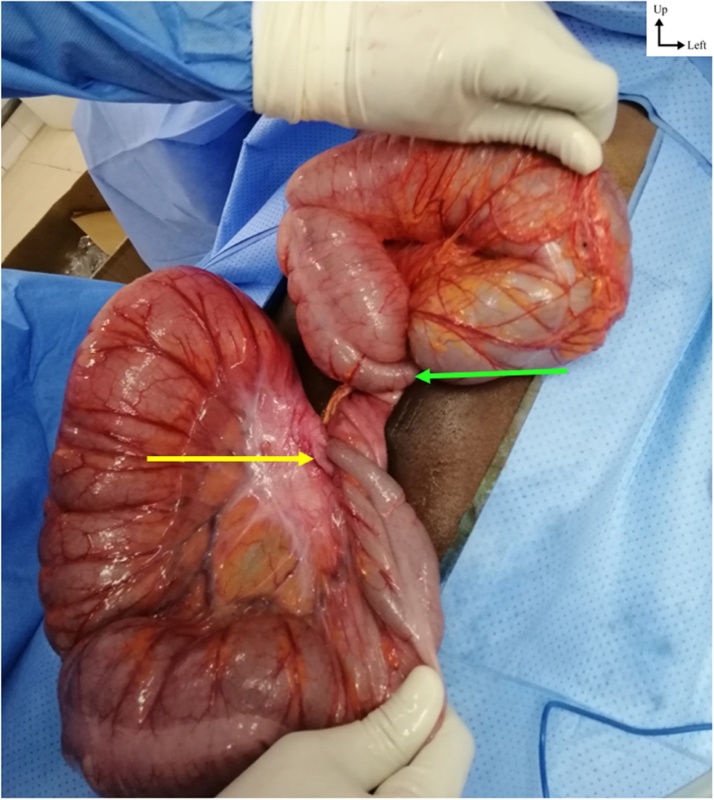
Intraoperative image showing a double volvulus of the sigmoid colon (yellow arrow) and the transverse colon (green arrow).

An exploratory laparotomy was performed a senior general surgeon. It showed sigmoid colon volvulus associated with synchronous transverse colon volvulus without bowel necrosis (Fig. 2). A left hemicolectomy with loop colostomy was performed. The post-operative course was uneventful. The patient was hospitalized in the General Surgery Department and the restoration of bowel continuity was done 3 weeks. With a follow-up of 5 months, no complication was noted and the patient mentioned any problem in his quality of life.

## 3 Discussion

A systematic review of the literature between 2000 and 2019 was performed. The search engines used were PubMed, Google scholar, and African Journal Online (AJOL). The search terms were “volvulus”, “sigmoid or pelvic”, “transverse”, “colon” ​​associated or not with the terms “simultaneous”, “synchronous”, “combined” and “double”. Only articles (clinical case, series of cases) written in English or French describing simultaneous volvulus of the sigmoid and transverse colon were included. Recurrence of volvulus after colonic resection were not included. In addition, a manual search of references from identified articles was performed to ensure that no article was inadvertently forgotten. The SCARE criteria was used to assess every article [1]. A total of 6 clinical cases were included. The parameters studied were: age, sex, diagnostic modalities, associated conditions, intraoperative findings and treatment. The different studies and their results are detailed in Table 1.

**Table 1:** Results.

References	Age (years)	Gender	Diagnostic modalities	Associated conditions	Intra operative findings	Treatment
Wisler et al. [8]	NA (Not available)	Male	Radiography	Chronic abdominal distension	No gangrene	Resection + end-transverse colostomy
Katsanos et al. [9]	83	Female	Radiography CT Scan Endoscopy	Ulcerative colitis	Megacolon with gangrene	Left hemicolectomy + transversectomy
McBrearty et al. [10]	63	Female	Radiography CT Scan	Constipation	No gangrene Sigmoid-transverse knot with unfixed left colon	Fixation of sigmoid and left colon + Coecostomy
Lianos et al. [11]	82	Female	Radiography CT Scan	Constipation	Megacolon Viablity (NA)	Total colectomy + Ileostomy
Hoseini et al. [12]	73	Female	Radiography	Constipation and Chronic abdominal distension	Megacolon with gangrene	Subtotal colectomy + Ileo-rectal anastomosis
Motsumi et al. [13]	26	Male	Radiography	NA	No gangrene	Subtotal colectomy + colostomy
Ndong et al. (2020)	74	Male	Radiography	Constipation	No gangrene	Left hemicolectomy + colostomy

Sigmoid colon volvulus is a relatively common cause of bowel obstruction. At the opposite, transverse colon volvulus first described in 1932 by Kallio, is uncommon (1–4% of cases) [14]. As a result, synchronic volvulus of the sigmoid and transverse colon is an even rarer situation [14]. Our review of the literature found only 6 clinical cases described in addition to our patient over 20 years (2000–2019).

The mean age of the patients was 66.8 years and all patients were over 60 years old except one who was 26 years old. Indeed, advanced age is a factor of exposure to constipation and intestinal hypomotility, which favor the onset of volvulus.

In colonic volvulus, a relative male predominance is described [14]. Which was not the case in our review where there were 4 women and 3 men.

The etiological factors of colon volvulus are relatively the same regardless of the site. The occurrence of simultaneous volvulus is caused by the same factors probably acting in concert. Indeed, several factors are incriminated and are of 3 types: anatomical, physiological, and congenital.

Anatomical factors described in the literature are those associated with abnormal mobility of the colon. These are mainly dolichocolon, whether or not associated with megacolon or Chilaiditi syndrome [14]. In three patients, there was associated megacolon and in one case unfixed left colon [[9], [10], [11], [12]].

Particularly in a case report, simultaneous volvulus was associated with ulcerative colitis [9]. This association with chronic inflammatory disease could be explained by the fact that chronic inflammation promotes fixation, twisting, and dilation of the intestine [9].

Physiological factors are represented by chronic constipation and/or overuse of laxatives leading to motility disturbances which can promote volvulus. In our review, 4 of 7 patients presented with chronic abdominal distension or chronic constipation [8,[10], [11], [12]]. The present case presented also in his history chronic constipation.

Among the congenital factors, there are intestinal malrotations which increase the capacity of the colon to twist on the axis of its meso because lack of fixation. Hirschsprung’s disease is also a congenital factor in volvulus, especially in children and adolescents. In one patient, there was an absence of fixation of the left colon causing the simultaneous sigmoid-transverse knot volvulus [12].

However, these different factors are often associated and have a synergistic effect in the increased mobility of the colon that causes its volvulus.

Clinically, symptoms of bowel obstruction were found in all patients with no particular pattern suggesting double volvulus. In fact, imaging remains essential, but the used modalities depend on surgical teams. In resources limited context, plain abdominal radiography is helpful. It was used in all the cases reported in our review. Indeed, the classic image of the coffee bean or the northern exposure or inverted U-shaped sign is not found if there is a simultaneous volvulus [15]. Rather, there is diffuse colonic distension which may require additional imaging explorations. Thus, the CT scan has better sensitivity and specificity in the diagnosis. It can show two concomitant whirl sign which can suggest a simultaneous volvulus. However, the CT scan is not available in all centers. It has only been used in three patients and has only suggested simultaneous volvulus in 2 cases [9,10]. In the third patient, it only visualized massive distension of the colon [11]. This explains why the preoperative diagnosis remains difficult and the simultaneous volvulus is often discovered during the surgery, as in 5 out of 7 patients [8,[11], [12], [13]].

Initial non-operative management with endoscopic derotation is the gold standard for sigmoid volvulus if there is no sign of necrosis or perforation. This treatment is secondarily associated with a colectomy preferably with immediate anastomosis [9]. Therefore, treatment for simultaneous volvulus is primarily surgical. In our review of the literature, surgical treatment was performed in all patients without any prior attempts of endoscopic derotation. But it should also be noted that the unavailability of endoscopy in some developing countries explains why surgery is preferred [16].

The surgical technique performed strongly depends on the existence of necrosis. The literature review noted that all the patients had a colonic resection. The choice between primary and delayed anastomosis varies according to the teams. In our review, 6 patients on 7 had delayed anastomosis. This can be explained by the fact that, with the emergency context and the high probability of ischemia, delayed anastomosis seems safer by reducing the risk of anastomotic leakage [16]. This is even more true when there are two sites of volvulus. Despite the rarity of this situation and the lack of randomized studies, delayed anastomosis seems to be the best option.

## 4 Conclusion

The occurrence of a simultaneous sigmoid and transverse colonic volvulus is an exceptional situation. The preoperative diagnosis is difficult even if the CT scan can show in some cases double whirl sign. There are no guidelines in the treatment and a tailored approach should be used for each patient. Therefore, colectomy with delayed anastomosis seems to be the best option.

## Declaration of Competing Interest

The authors report no declarations of interest.

## Funding

The authors declare they have received no funding for the preparation of this document.

## Ethical approval

The ethical committee of the hospital gave the agreement to report this case.

## Consent

“Written informed consent was obtained from the patient for publication of this case report and accompanying images. A copy of the written consent is available for review by the Editor-in-Chief of this journal on request.”

## Author contribution

Konaté Ibrahima, Abdourahmane Ndong and Adja Coumba Diallo these authors participated in the making and correction of this document. All authors agreed with the publication of the document.

## Registration of research studies

Researchregistry 5917.

## Guarantor

Abdourahmane Ndong.

## Provenance and peer review

Not commissioned, externally peer-reviewed.
